# The vibrational and configurational entropy of α-brass^☆^

**DOI:** 10.1016/j.jct.2013.11.012

**Published:** 2014-04

**Authors:** Artur Benisek, Edgar Dachs, Miralem Salihović, Aleksandar Paunovic, Maria E. Maier

**Affiliations:** Materialforschung und Physik, Universität Salzburg, Hellbrunnerstr. 34, A-5020 Salzburg, Austria

**Keywords:** Fcc Cu–Zn alloy, Excess heat capacity, Excess vibrational entropy, Excess configurational entropy, Calorimetry, Computer simulations

## Abstract

The heat capacities of two samples of a fcc Cu–Zn alloy with the composition CuZn15 and CuZn34 were measured from *T* = 5 K to 573 K using relaxation and differential scanning calorimetry. Below ∼90 K, they are characterised by negative excess heat capacities deviating from ideal mixing by up to −0.20 and −0.44 J · mol^−1^ · K^−1^ for CuZn15 and CuZn34, respectively. The excess heat capacities produce excess vibrational entropies, which are less negative compared to the excess entropy available from the literature. Since the literature entropy data contain both, the configurational and the vibrational part of the entropy, the difference is attributed to the excess configurational entropy. The thermodynamics of different short-range ordered samples was also investigated. The extent of the short-range order had no influence on the heat capacity below *T* = 300 K. Above *T* = 300 K, where the ordering changed during the measurement, the heat capacity depended strongly on the thermal history of the samples. From these data, the heat and entropy of ordering was calculated. The results on the vibrational entropy of this study were also used to test a relationship for estimating the excess vibrational entropy of mixing.

## Highlights

• The heat capacity of two α-brass samples was measured from *T* = 5 K to 300 K.
• Above *T* = 300 K, the ordering/disordering processes were investigated calorimetrically.
• The vibrational and configurational entropies of α-brass were calculated.
• A volume vs. bulk modulus approach describing the excess entropy was tested.

## Graphical abstract

**Figure:**
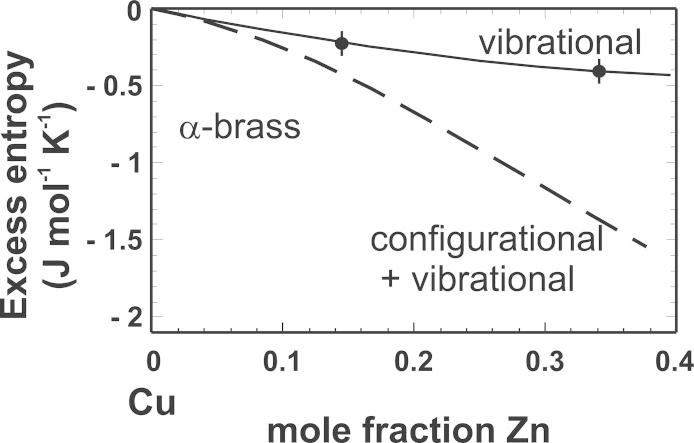

## 1 Introduction

The thermodynamics of copper-zinc alloys (brass) was subject of numerous investigations. Brass is characterised by an excess enthalpy and excess entropy of mixing, both of which are negative. The enthalpic data were measured by solution calorimetry e.g., [1–3] and based on chemical potential data calculated from phase equilibrium experiments e.g., [4–6], the excess entropy of mixing could be evaluated e.g., [7–9]. This excess entropy contains both, the vibrational and the configurational parts. The excess vibrational entropy, defined as the deviation from the entropy of a mechanical mixture of the end members A and B (i.e., $S_{\text{m}}^{\text{mechmix}}=X_{\text{A}}\;S_{\text{m}}^{\text{A}}+X_{\text{B}}\;S_{\text{m}}^{\text{B}}$), can be determined by measuring the low temperature heat capacity (5 to 300 K) versus composition behaviour. The determination of the excess configurational entropy, i.e., the excess entropy coming from non-random atomic distributions and defects, is much more difficult. Here, neutron scattering investigations together with computer simulations are normally used. If, however, reliable data of the total excess entropy (from enthalpic and chemical potential data) are available, the measurement of the excess vibrational entropy enables the determination of the excess configurational entropy simply by subtraction. Since configurational and vibrational entropies may have different temperature dependencies, it is worthwhile to separate the entropic effects. This is one aim of this study. Another aim is to deliver experimental data so that first principles studies can test their models on a disordered alloy, whose structural details (short-range order) depend on temperature.

Cu–Zn alloys in the Cu-rich compositional region (up to ∼38 mol% Zn) have the fcc disordered structure at high temperatures [10], known as α-brass. The atomic distribution, however, is not random. Different methods, e.g., neutron scattering [11], calorimetry [12], and first principles investigations [13] found that short-range order occurs in α-brass. A long range ordered Cu_3_Zn-phase as found in other 3:1 alloys (e.g., Cu_3_Au with L1_2_-structure or Al_3_Ti with DO_22_-structure) was never detected experimentally. This is probably due to its low transition temperature. First principles studies proposed either an ordered DO_23_-structure below 295 K [13] or an ordered L1_2_-structure below 288 K [14].

The excess vibrational entropy may have large effects on phase stability calculations (for a review, see for example [15,16]). For describing the excess vibrational entropy, a bond length versus bond stiffness interpretation [15] was proposed. According to this interpretation, changes in the stiffness of a chemical bond are mainly produced by changes of the bond lengths, which occur with compositional variations. Such a relation was found for the Pd-V and Ni–Al systems using first principles methods [17,18]. A substitution of an atom of different size produces both bond softening of the smaller atom and bond stiffening of the larger atom. However, one of both effects may dominate the vibrational behaviour of the solid solution giving rise to excess vibrational entropies. Recently, a simple relationship was presented [19] for estimating the maximum extent of the excess vibrational entropy ($\Delta_{\text{max}}S_{\text{m}}^{\text{exc}}$). The functionality of this relationship can be described by comparing the stiffness of the end members and distinguishing two different cases. Case 1: The larger end member is elastically stiffer. In this case, the atoms of the smaller end member have to enlarge their bond lengths in the solid solution to a high degree. Their bonds are strongly softened, which generates positive excess vibrational entropies. Case 2: The smaller end member is much stiffer. These conditions produce strongly compressed bonds of the atoms of the larger end member and in consequence negative excess vibrational entropies. To calculate $\Delta_{\text{max}}S_{\text{m}}^{\text{exc}}$, the relationship uses the differences of the end member volumes (Δ*V*_i,m_) and the differences of the end member bulk moduli (Δ*K*_i_), i.e.,

(1) $$\Delta_{\text{max}}S_{\text{m}}^{\text{exc}}/(\text{J}\cdot {\text{mol}}^{-1}\cdot {\text{K}}^{-1})=(\Delta V_{\text{i,m}}/(\text{J}\cdot {\text{mol}}^{-1}\cdot {\text{Pa}}^{-1})+\text{m}\Delta K_{\text{i}}/\text{Pa})\text{f.}$$

Based on data of silicate solid solutions and binary alloys, the parameters m and f of this equation were determined to be m/(J · mol^−1^ · Pa^−2^) = 1.089 · 10^−16^ and f/(Pa K^−1^) = 2.505 · 10^5^ (after converting the values of [20] into SI units). Δ*V*_i,m_ is defined to be positive whereas Δ*K*_i_ has a positive or negative value separating the above described cases. If the larger end member is elastically softer, then Δ*K*_i_ values are defined to be negative and vice versa. The behaviour of the excess vibrational entropy can for example be demonstrated by comparing some Mg–Ca substituted materials. As can be seen from table 1, the increasing volume mismatch does not necessarily generate increasing excess vibrational entropies. The increasing volume mismatch from (Mg,Ca)_3_Al_2_Si_3_O_12_ to (Mg,Ca)CO_3_ is compensated by increasing negative Δ*K*_i_ values. There are also solid solution systems, which are characterised by a low volume mismatch, but large negative Δ*K*_i_ values comparable to those of (Mg,Ca)CO_3_. This is the case for some metallic systems e.g., the Cu–Zn binary. Here, we expect negative excess vibrational entropies. Up to date, equation (1) was successfully applied to various silicate solid solutions [19,21,22], some binary alloys [20] and the NaCl–KCl binary [23]. The third aim of this paper is to test this relationship on α-brass.

## 2 Experimental methods

### 2.1 Cu–Zn samples

The CuZn15 sample was kindly provided by Austria buntmetall, Amstetten, Austria. The CuZn34 sample is from Alu-point, Harsum, Germany. The samples were examined by scanning electron microscopy detecting no compositional inhomogeneities. The compositions of the samples are listed in table 2. The structure of the samples was investigated by X-ray diffraction in order to check for the presence of the bcc-alloy. No bcc-reflections were found.

### 2.2 Relaxation calorimetry (PPMS)

Low-temperature heat capacities from 5 K to 300 K were measured using a commercially available relaxation calorimeter (heat capacity option of the PPMS by Quantum Design®). Pieces with ca. 4 × 4 × 0.3 mm (∼50 mg) were polished and mounted onto the calorimeter platform using Apiezon N grease. The measurements were repeated if the sample coupling, a measure of the quality of the thermal contact between sample and calorimeter platform, was lower than 90% (for details of the relaxation technique, see e.g., [32,33] and references therein). In such cases, the surface of the Cu–Zn pieces was reprocessed until a good sample coupling was achieved. It was found, however, that the PPMS measured heat capacities do not depend on the sample coupling, as it is the case when measuring oxide materials e.g., [34,35]. The accuracy of the PPMS heat capacities from *T* = 100 K to 300 K and the entropy at 298.15 K measured on single-crystal and sintered powder samples were found to be better than 0.5% [36].

### 2.3 Differential scanning calorimeter (DSC)

The heat capacity between *T* = 300 K and 573 K was measured using a power compensated Perkin Elmer Diamond DSC® on samples weighing ca. 150 mg. The DSC measurements were performed under a flow of Ar gas, with the calorimeter block kept at 250 K using a Perkin Elmer Intracooler. Each measurement consisted of a blank run with empty calorimeter chambers and a sample run, where the Cu–Zn sample and a pure Cu sample (with the same mass as the Cu–Zn sample and a purity of 99.8%) as reference material was placed into the calorimeter. The heat flow data (difference in heating power between the two chambers) were collected using a temperature scan (heating rate of 6 and 3 K/min) and isothermal periods of 3 min before and after the temperature scan. The heat flow versus temperature data from the sample run were shifted and rotated until the data of the isothermal periods agreed with those of the blank run (for details see e.g., [33]). The data from the blank run were then subtracted from those of the sample run to give the net heat flow of the sample. For calculating the heat capacity, the net heat flow data were finally divided by the heating rate and the mass of the sample. The accuracy of the DSC heat capacity data was determined to be better than 0.6% [33].

### 2.4 Evaluation of the raw heat capacity data

To calculate the vibrational entropy, the measured low temperature heat capacities were integrated numerically using an interpolation function of Mathematica® (interpolation order 2). The relative uncertainty of the entropy derived from the PPMS heat capacity data amounts to 0.2% for single-crystal and sintered powder samples as determined by a Monte Carlo technique in a previous study [37].

### 2.5 Calculations using density functional theory (DFT)

Quantum–mechanical calculations were based on the DFT plane wave pseudopotential approach implemented in the CASTEP code [38] included in the Materials Studio software from Accelrys®. The calculations were performed using the local density approximation [39]. Lattice dynamics calculations were based on the finite displacement approach implemented in CASTEP.

## 3 Results and discussion

### 3.1 Low-temperature heat capacities from 5 K to 300 K (PPMS)

**FIGURE 1:**
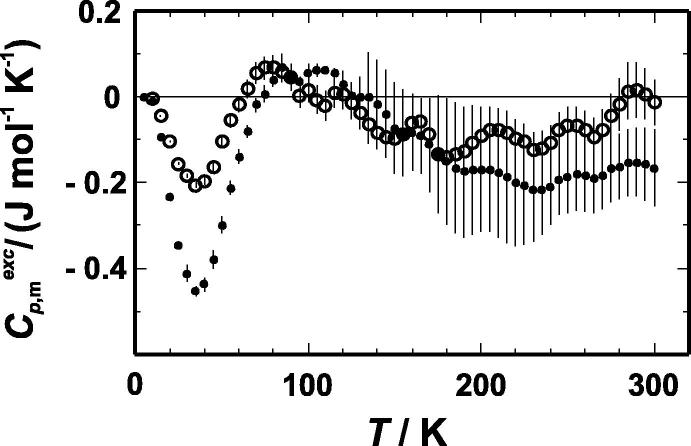
Molar excess heat capacity of mixing (*C_P_*_,m_^exc^) as function of temperature (*T*) for CuZn15 (○) and CuZn34 (●). Error bars represent one standard deviation.

The low-temperature heat capacities of the investigated samples are listed in table 3. The excess heat capacity (deviation from the behaviour of a mechanical mixture) was calculated using heat capacities of the end members from the literature [40–42]. It is plotted against temperature in figure 1 and shows a prominent negative peak at 40 K with maximum negative excess heat capacity values of −0.20 and −0.44 J · mol^−1^ · K^−1^ for CuZn15 and CuZn34, respectively. Between *T* = 90 K and 300 K, the excess heat capacity behaviour seems to be more or less ideal.

### 3.2 Heat capacities between *T* = 300 K and 573 K (DSC)

The heat capacities between *T* = 300 K and 573 K were found to depend strongly on the thermal history of the sample verifying the results of earlier investigations [12,43]. Prior to the measurements, the brass samples were, therefore, equilibrated at different temperatures which are given in table 4, and then quenched to room temperature. These equilibration experiments were repeated with an enlarged equilibration time until the heat capacity results converged (for final equilibration time see table 4).

**FIGURE 2:**
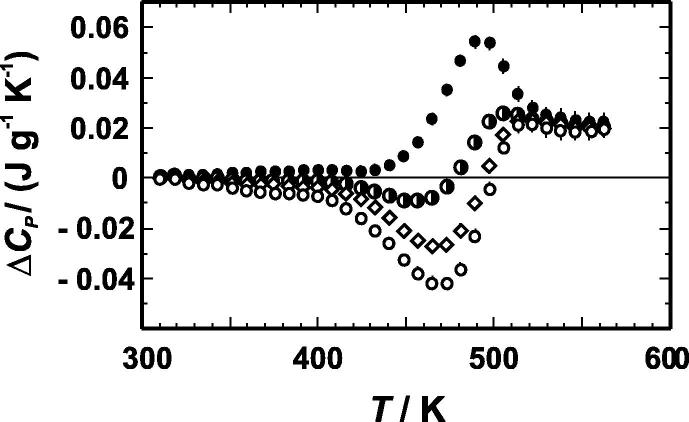
Heat capacity difference (Δ*C_P_*) between CuZn34 and pure copper as function of temperature (*T*). CuZn34 was equilibrated prior to measurements at temperatures of 413 K (●), 498 K (◑), 573 K (♢), and 673 K (○). Error bars represent one standard deviation and are mostly smaller than the symbols.

Figure 2 and table 4 present the heat capacity difference between the CuZn34 sample and pure copper. The differences of the heat capacities seen in figure 2 can be interpreted as heat capacities associated with short-range ordering/disordering ($\Delta C_{P}^{\mathrm{ord}}$). The sample quenched from 673 K shows a large negative peak at 470 K indicating energy release. Such an exothermic reaction can be attributed to an ordering process. Another sample was equilibrated at a low temperature of 413 K. Its Cu–Zn distribution is expected to be more ordered compared to the sample quenched from 673 K. The DSC measurements on the sample equilibrated at 413 K show accordingly a prominent positive peak at 490 K, where energy is absorbed. This energy absorption is due to a Cu–Zn disordering. Above ca. 530 K, the samples show similar behaviour independent from their thermal history, i.e., an energy of ca. 0.02 J · g^−1^ · K^−1^ is absorbed during heating, which can be attributed to a still ongoing disordering reaction above 530 K. The fact that all samples, independent of their thermal history, show a similar heat capacity behaviour above 530 K indicates that the distributions of Cu and Zn in the samples have reached their equilibrium values for the given temperatures (heating rate of 6 K/min). The peaks at lower temperatures arise from the energetic effects that result from a depletion of the non-equilibrium Cu–Zn distributions present in the samples at room temperature. The enthalpy changes due to ordering/disordering can be directly calculated from the heat capacities differences (figure 2) via the integration of Δ*C_P_ dT* from 300 to 573 K. The sample, which was equilibrated at 413 K for example, gives an enthalpy of disordering of 264 J · mol^−1^ (table 5). The sample, which was equilibrated at 573 K, undergoes first an ordering process followed by the disordering. The net enthalpy and entropy of ordering at 573 K of this sample is zero. This experimental result is to be expected, because the Cu–Zn distribution at the beginning of the experiment (frozen in at 573 K) should be the same as that at the end of the experiment (573 K). It confirms the suggestion made that the heat capacity differences seen in figure 2 are solely due to ordering/disordering processes. Using the enthalpy of ordering (Δ*H*^ord^) values of table 5, equilibrium $\Delta C_{P}^{\mathrm{ord}}$ values were first calculated by fitting the Δ*H*^ord^ − *T* values to a second order polynomial in *T* and differentiating this with respect to temperature. From the so calculated $\Delta C_{P}^{\mathrm{ord}}$, the entropy of ordering (Δ*S*^ord^) was then derived by integration of $\Delta C_{P}^{\mathrm{ord}}$/*T* over the temperature intervals listed in table 5.

The most ordered and most disordered samples were studied by low-temperature calorimetry, too. No heat capacity differences could be observed between *T* = 5 K and 300 K. The entropy of ordering/disordering derived from the DSC measurements can thus be associated solely to the change in the configurational entropy and does not contain any vibrational parts. To discuss these circumstances in more detail, let us first consider a sample, which was quenched from 573 K (marked with subscript 1). Its entropy at 573 K ($S_{1}^{573}$) is given by the following equation:

(2) $$S_{1}^{573}/\text{R}=S_{1}^{0}/\text{R}+\int_{0}^{573}C_{P\text{,m,}1}/\mathrm{TdT}/\text{R.}$$

Another sample whose atomic distribution was equilibrated at 413 K (marked with subscript 2) has the following entropy at 573 K:

(3) $$S_{2}^{573}/\text{R}=S_{2}^{0}/\text{R}+\int_{0}^{573}C_{P\text{,m,}2}/\mathrm{TdT}/\text{R.}$$

At 573 K, the entropy of both samples must be the same, because their Cu–Zn distribution is in equilibrium with this temperature (figure 2). The difference between $\int_{300}^{573}C_{P\text{,m,}1}/\mathrm{TdT}$ and $\int_{300}^{573}C_{P\text{,m,}2}/\mathrm{TdT}$ must, therefore, be equal to the difference between $S_{1}^{0}$ and $S_{2}^{0}$, because at temperatures below 300 K, there is no difference in *C_P_*_,m_. $S_{1}^{0}$ and $S_{2}^{0}$ are the configurational entropies at *T *= 0 K of sample 1 and 2 whose atomic distributions were frozen in at 573 and 413 K, respectively.

### 3.3 Excess entropy of mixing

The vibrational entropies of the CuZn15 and CuZn34 samples at 298.15 K are 34.17 (±0.07) and 35.63 (±0.07) J · mol^−1^ · K^−1^, respectively (table 6). These values are independent from the thermal history of the samples (Section 3.2). Comparing long range ordered with disordered samples in other metallic systems, distinct differences in the vibrational entropy were found e.g., [44–46]. It is possible that the change in vibrational entropy with ordering becomes effective only with long range ordering. However, this suggestion should be a subject of further studies.

**FIGURE 3:**
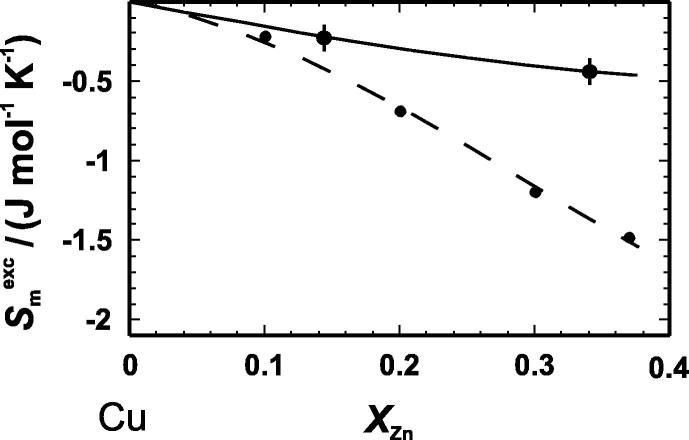
Molar excess entropy (*S*_m_^exc^) as function of zinc mole fraction (*X*_Zn_) at 573 K. The excess vibrational entropy (—) was measured in this study. The total excess entropy (– –) includes the vibrational and configurational parts and has been taken from [9], which is based on the work of [8]. Error bars represent one standard deviation.

Based on the vibrational entropy values of table 6, the excess vibrational entropy at *T *= 298.15 K was calculated to be −0.22 and −0.44 J · mol^−1^ · K^−1^ for the CuZn15 and CuZn34 samples, respectively. Applying a symmetric Margules mixing model to the data, the maximum extent of the excess vibrational entropy is obtained yielding $\Delta_{\text{max}}S_{\text{m}}^{\text{exc,calor}}$/(J · mol^−1^ · K^−1^) = −0.5. The excess vibrational entropies are generated mainly at low temperatures (below ∼90 K) and do not change at temperatures above 300 K (Section 3.2). The excess entropy values from the literature at 573 K [9, as the most recent data compilation] are much more negative compared to the excess vibrational entropy derived in this study (figure 3 and table 6). Since the literature excess entropy is derived from phase equilibrium experiments, it contains both, the vibrational and the configurational parts of the entropy. The difference between literature and our calorimetric excess entropy values must be, therefore, the excess configurational entropy at *T *= 573 K resulting in −0.20 and −0.92 J · mol^−1^ · K^−1^ for CuZn15 and CuZn34, respectively (table 6).

**FIGURE 4:**
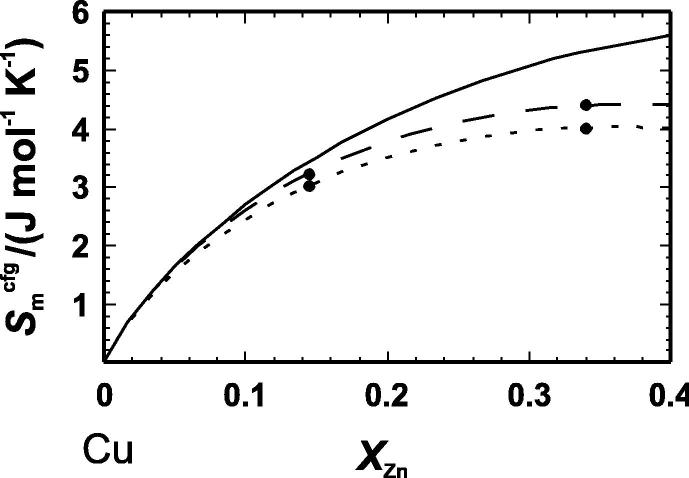
Molar configurational entropy (*S*_m_^cfg^) of α-brass as function of zinc mole fraction (*X*_Zn_) at 473 (· · ·) and 573 K (– –). These curves represent a Margules fit through the data points. The solid line (—) represents the configurational entropy of a randomly distributed binary system.

From the values of Δ*S*^ord^ determined by the DSC measurements and given in table 5, the temperature dependence of the excess configurational entropy can be calculated. At a temperature of ca. 500 K, it amounts to 0.0020 and 0.0030 J · mol^−1^ · K^−2^ for CuZn15 and CuZn34, respectively. From these values, the excess configurational entropy at 473 K was calculated (table 6). In figure 4, the configurational entropy of α-brass at 573 and 473 K is compared to that of a fully disordered Cu–Zn distribution demonstrating a significant deviation from it. The temperature dependence of the ordering enthalpy of our CuZn34 sample agrees well with an earlier study [12], investigating a CuZn31 sample.

### 3.4 Test of equation (1)

Equation (1) can be tested on the calorimetrically determined excess vibrational entropy values obtained in this study on the Cu–Zn binary. Based on volumes (*V*_m_) and bulk moduli (*K*) for copper and zinc from the literature [26,47], the obtained difference in end-member volumes is relatively small (Δ*V*_i,m_ = 2.05 · 10^−6^ m^3^), whereas the difference of the end-member bulk moduli has a large negative value (Δ*K*_i_ = −77.2 · 10^9^ Pa). The smaller end-member Cu is, thus, elastically much stiffer compared to Zn. Accordingly, strongly compressed Zn bonds and in consequence negative excess vibrational entropies can be expected. Using these data and equation (1), a $\Delta_{\text{max}}S_{\text{m}}^{\text{exc}}$ value of −1.59 J · mol^−1^ · K^−1^ is obtained, which is much more negative compared to the calorimetrically determined value ($\Delta_{\text{max}}S_{\text{m}}^{\text{exc,calor}}$/(J · mol^−1^ · K^−1^) = −0.5, Section 3.3).

The Cu–Zn system is characterised by structural changes. Copper and α-brass have the fcc-structure, whereas zinc crystallises in the hcp-lattice. The primitive cell of the fcc-structure contains only one atom having 3 acoustic vibrational modes. On the other hand, the primitive cell of the hcp-structure contains two crystallographic different sites, resulting in 3 acoustic and 3 optical modes. Zn in the hcp-structure may have a distinct different entropy compared to a hypothetical zinc crystallised in the fcc-structure. It is, therefore, possible that the structural differences between Cu and Zn are responsible for the disagreement in the above-mentioned $\Delta_{\text{max}}S_{\text{m}}^{\text{exc}}$ values, which can be tested using the density functional theory (DFT).

**FIGURE 5:**
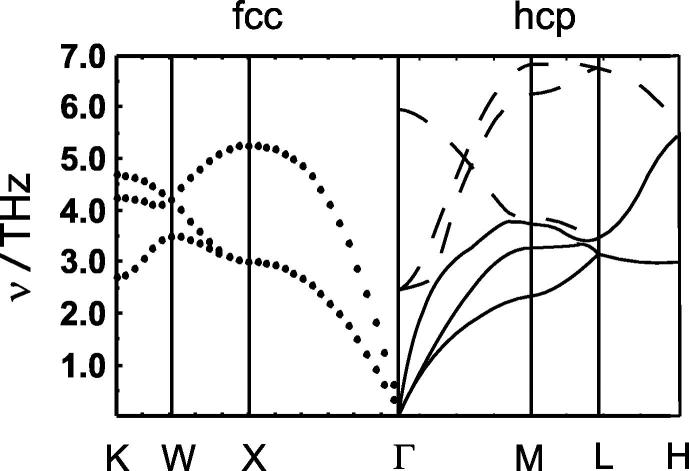
DFT-calculated dispersion relation for Zn. Solid (—) and broken (– –) lines are the acoustic and optical modes of the hcp-structure. The *dotted lines* (…) are the acoustic modes of a hypothetical fcc-structure.

**FIGURE 6:**
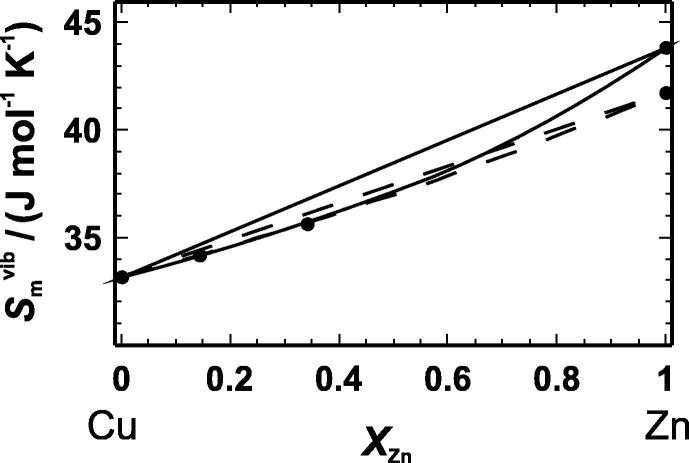
Molar vibrational entropy ($S_{\text{m}}^{\text{vib}}$) at 298.15 K. The broken lines (– –) represent $S_{\text{m}}^{\text{vib}}$ of the fcc–hcp binary with Cu and brass in the fcc- and Zn in the hcp-structure. The solid lines (—) represent $S_{\text{m}}^{\text{vib}}$ of a hypothetical binary, where all samples have the fcc-structure. The straight lines represent $S_{\text{m}}^{\text{vib}}$ of a mechanical mixture; the curved lines are a fit through the data using a Margules mixing model.

To define the entropy of a Cu–Zn system without any structural changes, the entropy difference between a hypothetical fcc-Zn and the hcp-Zn structure has to be calculated. Therefore, lattice dynamics calculations using the DFT plane wave pseudopotential approach of both structures were performed. The resulting dispersion relations are compared in figure 5. The mean frequency of the fcc-structure is lower compared to that of the hcp-structure and it seems that the absence of different crystallographic sites and in consequence the absence of the optical modes in the fcc-structure is responsible for this shift. The vibrational entropy of fcc-Zn is, therefore, higher than that of the hcp-structure. The entropy change of the hcp-fcc phase transition amounts to Δ_trans_*S*/(J · mol^−1^ · K^−1^) = 2.1 ± 0.5 depending on which pseudopotential was used. A new binary can, thus, be constructed, i.e., the fcc Cu–Zn binary. The excess vibrational entropies of this new binary are much larger (figure 6). Using again a Margules mixing model to calculate the maximum extent of the excess vibrational entropy of this fcc-binary results in $\Delta_{\text{max}}S_{\text{m}}^{\text{exc,calor,fcc}}$/(J · mol^−1^ · K^−1^) = −1.5. The volume and bulk modulus of the fcc-Zn structure has also to be recalculated using DFT calculations in order to give Δ*V*_i,m_ and Δ*K*_i_ values for the fcc Cu–Zn binary. The bonds in the fcc-Zn structure are slightly longer and softer compared to the hcp-Zn structure. Using DFT internally consistent *V*_m_ and *K* data for the fcc-binary and equation (1), the maximum excess vibrational entropy is $\Delta_{\text{max}}S_{\text{m}}^{\text{exc}}$/(J · mol^−1^ · K^−1^) = −1.63. This value is slightly more negative compared to that of the fcc-hcp binary ($\Delta_{\text{max}}S_{\text{m}}^{\text{exc}}$/(J · mol^−1^ · K^−1^) = −1.59). The volume effect of the hcp-fcc phase transition on the excess vibrational entropy is, thus, very low. Nevertheless, the $\Delta_{\text{max}}S_{\text{m}}^{\text{exc}}$ value calculated via equation (1) is in fairly good agreement with the calorimetric value ($\Delta_{\text{max}}S_{\text{m}}^{\text{exc,calor,fcc}}$/(J · mol^−1^ · K^−1^) = −1.5), suggesting that this equation is able to give a good estimate for the fcc Cu–Zn binary.

## 4 Conclusions

The study of the vibrational entropy of solid solutions enables the determination of the configurational entropy, if reliable enthalpic and phase equilibrium data exist.

The high mobility of the Cu and Zn atoms in α-brass makes an in situ calorimetric investigation of the ordering/disordering processes possible.

The negative excess vibrational entropy of the Cu–Zn alloy can be interpreted as follows: The end-member with the smaller volume (i.e., Cu) is elastically much stiffer compared to the end-member with the larger volume (i.e., Zn). The Cu atom will force, therefore, the Zn atom to fit to its size in the solid solution. The Zn bonds become, thus, much stiffer compared to the bonds in the pure phase producing the negative excess heat capacity of mixing. If the effect of the structural changes along the Cu–Zn binary is taken into account, equation (1) gives a good estimate for this system, too.

## Figures and Tables

**TABLE 1:** The maximum extent of the excess vibrational entropy ($\Delta_{\text{max}}S_{\text{m}}^{\text{exc}}$) as a function of the differences between end member volumes (Δ*V*_i,m_) and the differences between end member bulk moduli (Δ*K*_i_) for three Mg–Ca substituted materials. The values for $\Delta_{\text{max}}S_{\text{m}}^{\text{exc}}$ and Δ*V*_i,m_ are normalised to a substitution of one atom.

	Δ*V*_i,m_/(10^−5^ m^3^)	Δ*K*_i_/(10^9^ Pa)	$\Delta_{\text{max}}S_{\text{m}}^{\text{exc}}$/(J · mol^−1^ · K^−1^)
(Mg,Ca)_3_Al_2_Si_3_O_12_	0.40 [24]	−1 [24]	+1.0 [25]
(Mg,Ca)O	0.55 [26]	−50 [27,28]	∼0^a^
(Mg,Ca)CO_3_	0.88 [30]	−72 [30]	∼0 b

*a* A first principles study [29] proposed low vibrational effects when comparing the results with experimentally determined phase stabilities.

*b* A theoretical study [31] found agreement with experimental data without including vibrational effects.

**TABLE 2:** Electron microscope analyses in atomic%. The standard deviation is given in parentheses and refers to the last digit. The sample name represents weight%.

Sample	Cu	Zn
CuZn15	85.6 (2)	14.4 (2)
CuZn34	66.5 (2)	33.5 (2)

**TABLE 3:** Measured molar PPMS heat capacities (*C_p_*_,m_) of the Cu–Zn samples. The uncertainties in *T* and *C_p_*_,m_ are given as follows: *σ_T_* = ±*T*/K * (0.0007 + 9.4 * 10^−7 ^* *T*/K); *σ_Cp_*_,m_ = ±*C_p_*_,m_/(J · mol^−1^ · K^−1^) * (0.004 − 9.9 * 10^−6^ * *T*/K).

CuZn15		CuZn34	
T/K	*C_p_*_,m_/(J · mol^−1^ · K^−1^)	*T*/K	*C_p_*_,m_/(J · mol^−1^ · K^−1^)
5.049	0.0112	5.049	0.0125
5.398	0.0132	5.418	0.0149
5.790	0.0155	5.809	0.0178
6.206	0.0184	6.224	0.0214
6.665	0.0219	6.683	0.0258
7.139	0.0262	7.149	0.0311
7.652	0.0313	7.661	0.0376
8.201	0.0377	8.210	0.0457
8.790	0.0456	8.800	0.0558
9.421	0.0551	9.429	0.0681
10.10	0.0671	10.11	0.0836
10.82	0.0819	10.83	0.1025
11.60	0.1003	11.62	0.1264
12.44	0.1236	12.45	0.1561
13.33	0.1528	13.35	0.1931
14.29	0.1888	14.31	0.2390
15.32	0.2348	15.33	0.2975
16.42	0.2927	16.44	0.3709
17.59	0.3656	17.62	0.4624
18.86	0.4581	18.88	0.5770
20.23	0.5754	20.23	0.7193
21.68	0.7202	21.69	0.8964
23.24	0.9012	23.24	1.1161
24.91	1.1249	24.91	1.3828
26.70	1.3919	26.70	1.6993
28.62	1.7038	28.62	2.0747
30.68	2.1191	30.68	2.5245
32.89	2.5556	32.89	3.0366
35.26	3.0729	35.25	3.6204
37.80	3.6666	37.79	4.2765
40.52	4.3368	40.51	5.0081
43.43	5.0730	43.42	5.7991
46.55	5.8826	46.55	6.6547
49.91	6.7616	49.90	7.5628
53.50	7.6852	53.49	8.5199
57.35	8.6556	57.35	9.5109
61.48	9.6552	61.47	10.514
65.90	10.682	65.89	11.531
70.65	11.724	70.64	12.559
75.72	12.735	75.71	13.539
81.17	13.734	81.15	14.528
87.00	14.707	86.99	15.464
93.25	15.586	93.22	16.326
99.95	16.509	99.92	17.215
107.14	17.323	107.11	18.031
114.87	18.177	114.82	18.799
123.12	18.920	123.05	19.467
131.97	19.590	131.94	20.139
141.46	20.217	141.43	20.740
151.65	20.819	151.61	21.254
162.55	21.420	162.50	21.783
174.26	21.846	174.21	22.203
186.86	22.312	186.76	22.603
200.28	22.772	200.17	22.997
214.67	23.155	214.58	23.330
230.11	23.455	230.00	23.610
246.59	23.820	246.53	23.932
264.31	24.070	264.22	24.182
283.23	24.412	283.20	24.446
303.64	24.605	303.52	24.647

**TABLE 4:** Heat capacity difference (Δ*C_P_*) of CuZn34 due to Cu−Zn ordering/disordering. Prior to measurements, the sample was equilibrated at different temperatures (*T*^eq^). The equilibration time is also given (*t*^eq^). The numbers in parentheses represent one standard deviation and refer to the last digit. The uncertainty in the temperature was estimated to be ±1 K.

*T*^eq^/K	413	498	573	673
*t*^eq^/h	360	2	0.2	0.2
*T*/K	Δ*C_P_*/(J · g^−1^ · K^−1^)	Δ*C_P_*/(J · g^−1^ · K^−1^)	Δ*C_P_*/(J · g^−1^ · K^−1^)	Δ*C_P_*/(J · g^−1^ · K^−1^)
310.3	0.000 (1)	0.001 (1)	0.001 (1)	0.000 (1)
318.4	0.000 (1)	0.000 (1)	0.001 (1)	−0.001 (1)
326.5	0.001 (1)	0.000 (1)	0.000 (1)	−0.002 (1)
334.7	0.001 (1)	0.000 (1)	0.000 (1)	−0.003 (1)
342.9	0.001 (1)	0.000 (1)	0.000 (1)	−0.003 (1)
351.0	0.002 (1)	−0.001 (1)	−0.001 (1)	−0.004 (1)
359.2	0.002 (1)	−0.001 (2)	−0.001 (1)	−0.005 (1)
367.3	0.003 (2)	−0.001 (2)	−0.001 (2)	−0.005 (2)
375.5	0.003 (2)	−0.001 (2)	−0.001 (2)	−0.006 (2)
383.6	0.002 (2)	−0.001 (2)	−0.002 (2)	−0.006 (2)
391.7	0.003 (2)	−0.001 (2)	−0.003 (2)	−0.007 (2)
399.9	0.003 (2)	−0.001 (2)	−0.004 (2)	−0.007 (2)
408.0	0.003 (2)	−0.002 (2)	−0.005 (2)	−0.009 (2)
416.2	0.003 (2)	−0.002 (2)	−0.006 (2)	−0.012 (2)
424.3	0.003 (2)	−0.004 (2)	−0.008 (2)	−0.016 (2)
432.4	0.004 (2)	−0.005 (2)	−0.012 (2)	−0.021 (2)
440.5	0.005 (2)	−0.007 (2)	−0.016 (2)	−0.026 (2)
448.7	0.009 (2)	−0.009 (2)	−0.021 (2)	−0.032 (2)
456.8	0.014 (2)	−0.009 (3)	−0.025 (2)	−0.038 (3)
464.9	0.023 (3)	−0.008 (3)	−0.027 (3)	−0.042 (3)
473.0	0.035 (3)	−0.003 (3)	−0.027 (3)	−0.042 (3)
481.1	0.047 (3)	0.005 (3)	−0.021 (3)	−0.036 (3)
489.2	0.054 (3)	0.014 (3)	−0.010 (3)	−0.023 (3)
497.4	0.054 (3)	0.022 (3)	0.005 (3)	−0.004 (3)
505.5	0.044 (3)	0.026 (3)	0.017 (3)	0.012 (3)
513.6	0.034 (3)	0.025 (3)	0.023 (3)	0.021 (3)
521.7	0.028 (3)	0.023 (3)	0.023 (3)	0.021 (3)
529.8	0.025 (3)	0.022 (3)	0.022 (3)	0.019 (3)
537.9	0.024 (3)	0.022 (3)	0.022 (3)	0.018 (3)
546.0	0.023 (3)	0.022 (3)	0.021 (3)	0.018 (3)
554.1	0.023 (3)	0.022 (4)	0.021 (3)	0.019 (4)
562.2	0.023 (4)	0.023 (4)	0.020 (4)	0.020 (4)

**TABLE 5:** Enthalpy and entropy of ordering (Δ*H*^ord^, Δ*S*^ord^) for the CuZn34 and CuZn15 samples. Positive values correspond to enthalpy and entropy of disordering, whereas negative values correspond to enthalpy and entropy of ordering. Numbers in parentheses are estimated uncertainties and refer to the last digit.

CuZn34	Δ*H*^ord^/(J · mol^−1^)	Δ*S*^ord^/(J · mol^−1^ · K^−1^)
From 413 K to 573 K	264 (2)	0.56 (2)
From 498 K to 573 K	94 (2)	0.20 (2)
From 673 K to 573 K	−100 (2)	−0.15 (2)
CuZn15		
From 473 K to 573 K	103 (5)	0.20 (5)

**TABLE 6:** Molar vibrational entropy at *T *= 298.15 K ($S_{\text{m}}^{298.15-0}$), the excess vibrational entropy ($\Delta S_{\text{vib,m}}^{\text{exc}}$), the excess entropy from literature at 573 K ($\Delta_{573}S_{\text{lit,m}}^{\text{exc}}$) and the excess configurational entropy at 573 K ($\Delta_{573}S_{\text{cfg,m}}^{\text{exc}}$) and 473 K ($\Delta_{473}S_{\text{cfg,m}}^{\text{exc}}$).

Sample	$S_{m}^{298.15-0}$/(J · mol^−1^ · K^−1^)	$\Delta S_{\text{vib,m}}^{\text{exc}}$/(J · mol^−1^ · K^−1^)	$\Delta_{573}S_{\text{lit,m}}^{\text{exc}}$/(J · mol^−1^ · K^−1^)	$\Delta_{573}S_{\text{cfg,m}}^{\text{exc}}$/(J · mol^−1^ · K^−1^)	$\Delta_{473}S_{\text{cfg,m}}^{\text{exc}}$/(J mol^−1^ K^−1^)
Cu	33.164 a	0	0	0	0
CuZn15	34.17 ± 0.07	−0.22 ± 0.1	−0.42 b	−0.20	−0.40
CuZn34	35.63 ± 0.07	−0.44 ± 0.1	−1.36 b	−0.92	−1.22
Zn	41.717 a	0	0	0	0

*a* JANAF-tables [42].

*b* Reference [9].
